# Supplementary material for: Analysis of the Maximum Efficiency and the Maximum Net Power as Objective Functions for Organic Rankine Cycles Optimization
Source: Entropy (Basel). 2023 May 31;25(6):882. doi: 10.3390/e25060882 (PMC10297069; doi:10.3390/e25060882)
Supplement: Supplementary file 1 [file entropy-25-00882-s001.zip › entropy-2355143-supplementary.pdf]

Analysis of the maximum efficiency and the maximum net power as objective functions for organic Rankine cycles optimization

Supplementary material

Johan González[a], José Matías Garrido[b], Héctor Quinteros-Lama[a]

[a]*Departamento de Tecnologías Industriales, Faculty of Engineering, Universidad de Talca. Merced 437, Curicó, Chile*
[b]*Departamento de Ingeniería Química, Faculty of Engineering, Universidad de Concepción, Concepción, Chile*

S1. Generalities of geometrical an optimizing functions

The projection of the β-function together with the ψ-function is displayed in Fig. S1. The figure shows the parametric map where the ψ-function[1–3], in black, and the β-function, in crimson, are displayed. Point (A) represents the minimum value of ψ-function, i.e., the isentropic behavior, while Point (B) is the minimum values of β-function, i.e., the first optimum expansion of a dry fluid.

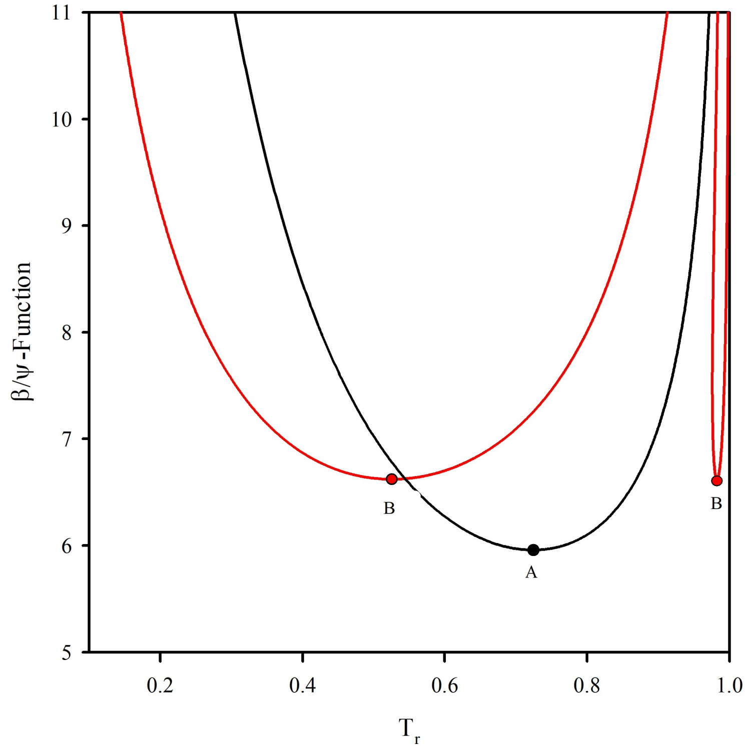

Figure S1: The ψ-function in black and the β-function in crimson as predicted by the van der Waals EOS at different values of heat capacities.

S1.1. The ψ-function

The ψ-function represent the geometrical features of the entropy curve in the temperature vs entropy projection. The details of the ψ-functions can be found in previous works for pure compounds[1] and mixtures[4]. In Figure S1, it can be observed that ψ is a parabolic convex function with a sharp minimum at Point (A). Moreover, it can be seen that the fluids necessarily exhibit wet behavior at low temperatures, the latter condition that may be masked by the freezing point ($T_r = 0.2$) and in the vicinity of the critical point. In Fig. S1, it can be observed that the ψ-function is a parabolic convex function with a sharp minimum at Point (A). Moreover, it

can be seen that fluids exhibit wet behavior at low temperatures, which may be masked by the freezing point and in the vicinity of the critical point. In the work[1], the area above the ψ-function is a dry region and the area below the ψ-function is a wet region. On the one hand, any C_P^{i}/R value of the fluid that is below of ψ-function, presents wet behavior along the whole saturation range. On the other hand, any C_P^{i}/R value of the fluid that is above of ψ-function, presents dry behavior along the whole saturation range. Concerning Point (A), it can be said that every molecule with C_P^{i}/R minor than Point (A) over all the saturation temperature range will unequivocally exhibit wet behavior.

S1.2. The β-function

The β-function shows the optimal operation conditions if the expansion is placed within the saturated vapor limit. The details of the β-functions can be found in previous work[3]. In the β-function, it can be seen in two different lines; a low-temperature line, representing the optimal temperature of the condenser, and a high-temperature line, representing the optimal condition of the boiler under the above conditions. It is an evident fact that no temperature for the condenser can be reached since the solidification of the species. For the aforementioned reason, the calculation of both functions was performed until $T_r = 0.2$, which is a temperature where most of the compounds are in the solid state, and hence outside the scope of this analysis.

Another interesting observation in Fig. S1 is that two branches emerge from Points (B). Both lines render optimum conditions but of different natures. The crimson line is where the feasible optimum lies, while the light line is also an optimum but non-desirable one. The aforementioned optimum lies between a near-critical temperature for the boiler and a temperature higher than the temperature where the entropy fulfills the constraints for ψ-function. Therefore, an ORC runs entirely in the interior of the saturation curve and is intrinsically wet.

As mentioned in Fig. S1, Point (A) is the isentropic behavior, while Point (B) is the first point where a dry-expansion optimum point can be obtained. From this analysis, considering a van der Waals fluid, the first conclusion is that a merely isentropic fluid is not enough to ensure a good performance in an ORC cycle, given that both behaviors are not connected. Other inferences can be obtained from a simple observation of Fig. S1. Firstly, despite the presence of a minimum, the primary behavior of the optimal efficiency is such that the complexity of the compound increase also increases the difference between the required boiler temperature and the temperature of the condenser, which in common cases has obvious technical limitations. Secondly, the range where the optimum boiler temperature lies is a narrow zone compared with the range of the condenser. The aforementioned fact carries that the temperature variations in the energy source of the boiler must be restricted. The above is important, particularly in utilizing low-quality heat such as geothermal, secondary, and other renewable sources.

Along with the above, an essential deduction is seen in Fig. S1, particularly in the high-temperature area. The optimum temperature for the boiler always lies at higher temperatures than the ψ-function. Therefore, there is not possible a perfectly dry expansion in an optimal condition.

References

(1) Garrido, J. M.; Quinteros-Lama, H.; Mejía, A.; Wisniak, J.; Segura, H. A rigorous approach for predicting the slope and curvature of the temperature-entropy saturation boundary of pure fluids. *Energy* **2012**, *45*, 888–899.

(2) Imre, A. R.; Kustán, R.; Groniewsky, A. Thermodynamic selection of the optimal working fluid for organic Rankine cycles. *Energies* **2019**, *12*, 1–15.

(3) González, J.; Llovell, F.; Garrido, J. M.; Quinteros-Lama, H. A rigorous approach for characterising the limiting optimal efficiency of working fluids in organic Rankine cycles. *Energy* **2022**, *254*, 124191.

(4) Albornoz, J.; Mejía, A.; Quinteros-Lama, H.; Garrido, J. M. A rigorous and accurate approach for predicting the wet-to-dry transition for working mixtures in organic Rankine cycles. *Energy* **2018**, *156*, 509 – 519.
